# Therapeutic plasma exchange for anti-glomerular basement membrane disease with dialysis-dependent kidney failure without diffuse alveolar hemorrhage

**DOI:** 10.1007/s40620-023-01695-9

**Published:** 2023-06-24

**Authors:** Hideaki Watanabe, Hayato Yamana, Akira Okada, Hiroki Matsui, Kiyohide Fushimi, Hideo Yasunaga

**Affiliations:** 1 Department of Clinical Epidemiology and Health Economics, The University of Tokyo, 7-3-1, Hongo, Bunkyo, Tokyo, 1130033 Japan; 2 Data Science Center, Jichi Medical University, Shimotsuke, Japan; 3 Department of Prevention of Diabetes and Lifestyle-Related Diseases, Graduate School of Medicine, The University of Tokyo, Tokyo, Japan; 4 Department of Health Policy and Informatics, Tokyo Medical and Dental University, Tokyo, Japan

**Keywords:** Anti-glomerular basement membrane disease, Therapeutic plasma exchange, Clinical epidemiology, In-hospital mortality, AKI

## Abstract

**Background:**

Anti-glomerular basement membrane (anti-GBM) disease is treated with immunosuppressive medications and plasma exchange. However, whether plasma exchange, in addition to pulse glucocorticoid therapy, would benefit patients with anti-GBM disease with dialysis-dependent kidney failure without diffuse alveolar hemorrhage remains unclear.

**Methods:**

Using the Japanese Diagnosis Procedure Combination database, we identified patients diagnosed with anti-GBM disease with dialysis-dependent kidney failure and without diffuse alveolar hemorrhage from July 2010 to March 2020. We compared in-hospital mortality within 10 days of hospitalization between patients who received therapeutic plasma exchange in addition to pulse glucocorticoid therapy and those who received pulse glucocorticoid therapy alone. Overlap weighting based on propensity score was performed to adjust for potential confounders.

**Results:**

We identified 207 eligible patients; 168 patients received therapeutic plasma exchange plus pulse glucocorticoid therapy, while 39 patients received pulse glucocorticoid therapy alone. The mean dose of therapeutic plasma exchange was 52.2 ml/kg/day of albumin and/or fresh frozen plasma. Therapeutic plasma exchange in addition to pulse glucocorticoid therapy was associated with a lower in-hospital mortality risk in the unweighted (10.7% versus 28.2%; risk difference, 17.5%; 95% confidence interval, 2.6–32.4%; *P* = 0.02) and weighted analyses (11.5% versus 28.4%; risk difference, 17.0%; 95% confidence interval, 1.5–32.5%; *P* = 0.03) than pulse glucocorticoid therapy alone.

**Conclusions:**

This retrospective cohort study using a national database suggests that therapeutic plasma exchange may improve the in-hospital prognosis of anti-GBM disease with dialysis-dependent kidney failure and without diffuse alveolar hemorrhage.

**Graphical abstract:**

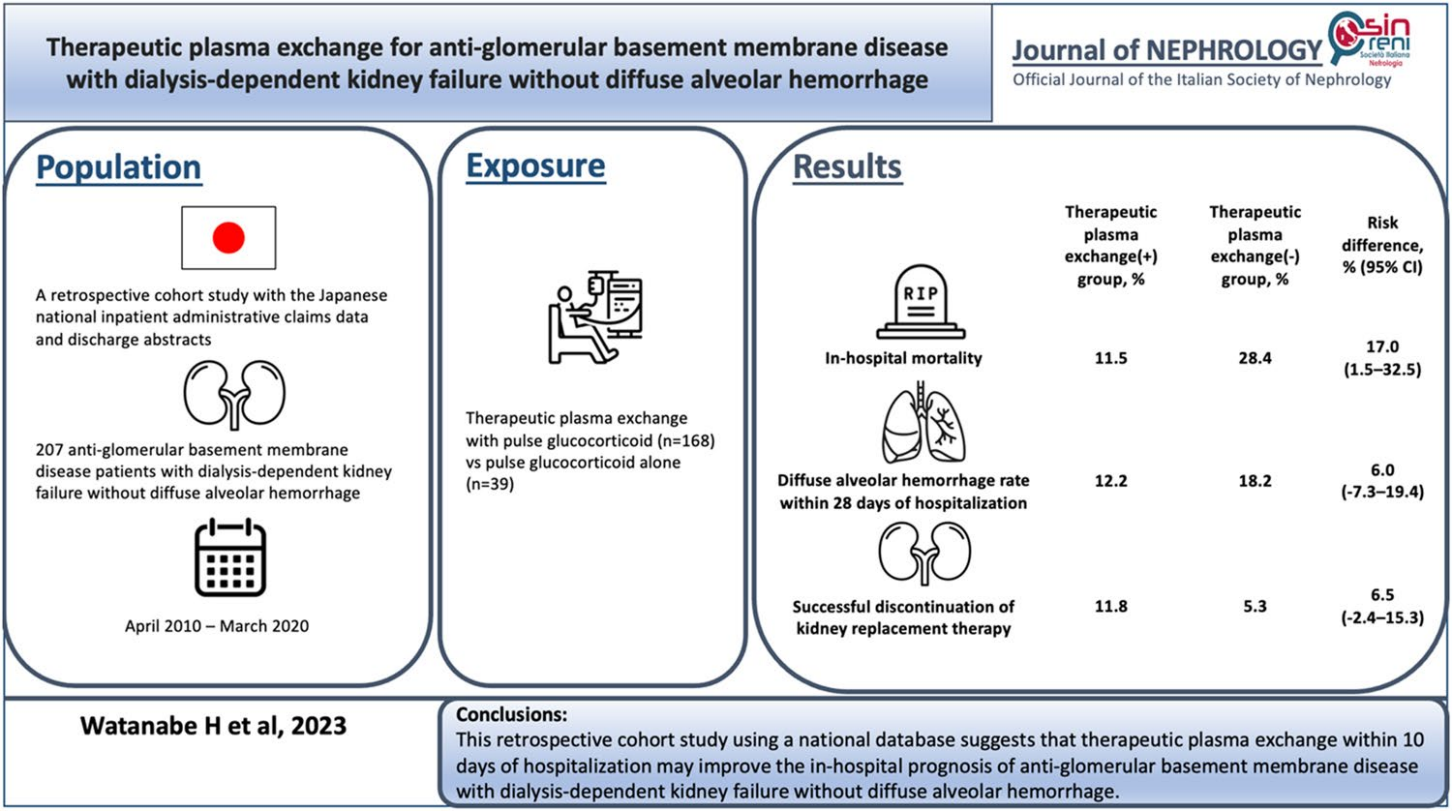

**Supplementary Information:**

The online version contains supplementary material available at 10.1007/s40620-023-01695-9.

## Introduction

Anti-glomerular basement membrane (anti-GBM) disease is a rare small-vessel vasculitis that damages glomerular capillaries, pulmonary capillaries, or both. Anti-GBM disease is estimated to occur in fewer than two individuals per million population per year [1]. Approximately 90% of patients with anti-GBM disease present with rapidly progressive glomerulonephritis, 25–65% with concomitant alveolar hemorrhage, and 6% with isolated pulmonary hemorrhage [2, 3].

Therapeutic plasma exchange (TPE) plus immunosuppressive therapy is the primary treatment for anti-GBM disease. Although most studies have been descriptive, single-armed, case series, or case reports [4–7], one single-center, single-armed retrospective study showed survival improvement in patients treated with TPE, prednisolone, and cyclophosphamide compared to nearly 100% mortality in the untreated historical cohort [8]. While a randomized study of 17 patients suggested that TPE plus immunosuppressive therapy might improve renal outcomes [9], large-scale randomized controlled trials have yet to be conducted. The American Society for Apheresis 2019 guidelines and Kidney Disease Improving Global Outcomes 2021 guidelines recommend treating patients with diffuse alveolar hemorrhage and/or dialysis-independent acute kidney injury (AKI) with TPE plus immunosuppressive therapy (grade 1, quality B to C) [10].

Notably, less evidence is available for patients with dialysis-dependent AKI without diffuse alveolar hemorrhage as only case reports or case series have been reported in this patient group [8, 11]. The American Society for Apheresis 2019 guidelines reviewed the level of evidence for TPE as grade 2 and quality B in dialysis-dependent AKI without diffuse alveolar hemorrhage. Therefore, whether the addition of TPE to standard immunosuppressive therapy would benefit patients remains unclear.

We aimed to evaluate the effectiveness of TPE in addition to pulse glucocorticoid therapy in patients with anti-GBM disease with dialysis-dependent AKI without diffuse alveolar hemorrhage, using a nationwide inpatient database in Japan.

## Methods

### Data source

We used the Diagnosis Procedure Combination database—a national inpatient database of administrative claims data and discharge abstracts in Japan. Details of the database are described elsewhere [12]. The database contains the following information: unique hospital identifiers; sex; age; height and body weight; smoking status; level of consciousness on admission and discharge based on the Japan Coma Scale; activities of daily living on admission and discharge recorded as the Barthel Index; main diagnosis, admission-necessitating diagnosis, comorbidities on admission, and in-hospital complications recorded using the International Classification of Diseases, 10th revision codes and text data in Japanese; performed in-hospital procedures; and daily dosages of drugs and devices. The Japan Coma Scale is reported to correlate well with the Glasgow Coma Scale [13].

The study was approved by the Institutional Review Board of the Graduate School of Medicine, the University of Tokyo. Because of the anonymous character of the data, the requirement for informed consent was waived.

### Study design and population

We extracted data on patients aged 20 years or older who were admitted between 1 July, 2010 and 31, March 2020. Cases were eligible when a diagnosis of anti-GBM disease or Goodpasture disease was recorded in the main diagnosis. When multiple eligible hospitalizations by the same patient occurred, we selected the first one. We specified the following exclusion criteria using data obtained within the first 10 days of hospitalization: anti-GBM antibody not tested, kidney replacement therapy (KRT) not started, and apparent diffuse alveolar hemorrhage (hemoptysis, hemorrhage from other sites in the respiratory passage, or abnormal findings in specimens from respiratory organs and thorax; implementation of endotracheal intubation, mechanical ventilator, or tracheostomy). We excluded patients discharged within 10 days of hospitalization to prevent immortal time bias [14] and patients who did not receive pulse glucocorticoid therapy (≥ 1500 mg of total prednisone-converted glucocorticoid in 10 days) within 10 days of hospitalization.

### Study outcomes and variables

We obtained the following information: age, sex, body mass index (BMI), Barthel Index on admission, smoking status, whether the institution was an academic hospital or not, Japan Coma Scale on admission, and Charlson Comorbidity Index. Additionally, we identified the following comorbidities using the respective International Classification of Diseases and Related Health Problems, 10th revision codes: diabetes mellitus (E10-E14), hypertension (I10, I11.0, I11.9, I12.0, I12.9, I13.9, I15.0-I15.2, I15.9), dyslipidemia (E78.0-E78.5), anti-neutrophil cytoplasmic antibody (ANCA) positivity (M30.1, M31.3, M31.7, M31.8, N01.7 with text data denoting ANCA-positive diseases in Japanese), and sepsis (Online Resource 1). The use of TPE, vasopressors (epinephrine, norepinephrine, dopamine, dobutamine, isoprenaline, ivabradine, etilefrine, olprinone, colforsin, denopamine, vasopressin, pimobendane, phenylephrine, bucladesine, vesnarinone, milrinone, ubidecarenone, or atropine), and red blood cell transfusion within 10 days of hospitalization was also identified. The database records the 10 components of the Barthel Index: feeding, bathing, grooming, dressing, bowels, bladder, toilet, transfers, mobility, and stairs. The total obtainable Barthel Index score is 100; however, considering the possibility of anuria in patients undergoing hemodialysis, we excluded the score for urination and considered 90 as the total score. As used in a previous article, we grouped patients as being physically dependent or independent, using a cut-off value of 90% of the total score [15]. The Charlson Comorbidity Index was calculated as described previously [16].

The primary outcome was in-hospital mortality. We also analyzed the following secondary outcomes: 28-day in-hospital mortality, 60-day in-hospital mortality, diffuse alveolar hemorrhage within 28 days of hospitalization, and successful discontinuation of KRT during hospitalization. Given no gold standard parameter when deciding on the discontinuation of KRT during AKI recovery [17], we defined successful discontinuation of KRT as being discharged alive after not having received KRT for more than 4 days.

### Statistical analyses

First, we summarized the numbers of patients who received TPE, cyclophosphamide, and rituximab—in addition to pulse glucocorticoid therapy. Thereafter, we excluded patients who received cyclophosphamide or rituximab within 10 days of hospitalization due to the small number of patients involved. We evaluated the mean dose and the total number of in-hospital administration days of TPE. Therapeutic plasma exchange dose was calculated using the average daily amount (ml/kg) of albumin and/or fresh frozen plasma. We then conducted the primary analysis that compared the group that was administered TPE within 10 days after admission in addition to pulse glucocorticoid therapy and the group that was administered pulse glucocorticoid therapy alone. We used overlap weights based on the propensity score to balance background characteristics between the two groups [18, 19]. Overlap weights have been shown to outmatch the inverse probability of treatment weighting, in terms of bias reduction [20]. The propensity score was estimated using logistic regression analysis with the receipt of TPE as the dependent variable. The following independent variables were specified: age (20–39, 40–59, 60–79, or ≥ 80 years old), sex, BMI (< 18.5, 18.5–24.9, 25.0–29.9, or ≥ 30.0 kg/m^2^), Barthel Index score (≥ 81 or < 81), smoking status (current/past smoker or not), academic hospital, Japan Coma Scale (consciousness clear or not), Charlson Comorbidity Index, presence of diabetes mellitus, hypertension, and/or dyslipidemia, ANCA positivity, use of vasopressors, and packed red blood cell transfusion. We calculated the c-statistics of the propensity score to assess its ability to differentiate the two groups. The estimated propensity score was then used for deriving overlap weights, defined as 1—the propensity score for patients who underwent TPE and the propensity score for those who did not. We compared patient backgrounds between the two groups before and after weighting. Standardized differences were used to evaluate the differences between groups, and an absolute standardized difference of > 10% indicated imbalance [21]. Finally, we performed weighted generalized linear model analyses with binomial distribution and log link function to obtain risk differences for the outcomes.

We performed sensitivity analyses by changing the timing to include the patients from 10 days of hospitalization to 5 or 15 days of hospitalization. We also conducted a complete case analysis for the primary outcome; that is, patients with missing data were excluded from this analysis. Moreover, we limited patients to those who received 3000 mg of glucocorticoids within 10 days of hospitalization. Finally, we conducted an analysis that included patients who received cyclophosphamide or rituximab, in addition to pulse glucocorticoid therapy. We performed three additional weighted survival time analyses: Cox regression analysis for in-hospital mortality, and competing risks analyses for death and successful discontinuation of KRT that considered one another as the competing risk. The overlap weights calculated in the main analysis were also used in these analyses. All statistical analyses were performed using Stata/SE version 17.0 software (StataCorp, College Station, TX). We used a two-tailed significance level of *P* < 0.05.

## Results

### Study population

**Fig. 1:**
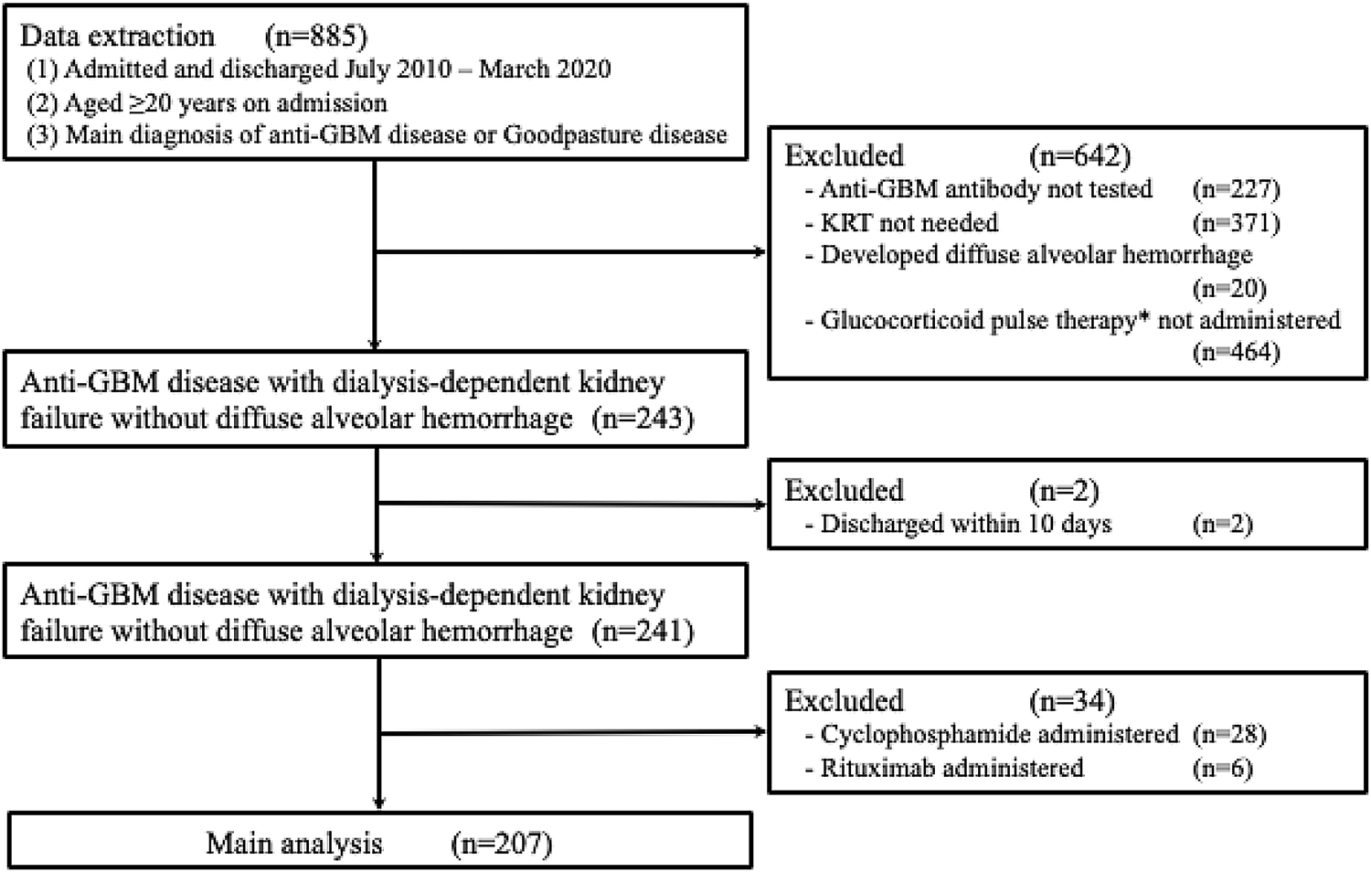
Selection of patients with anti-GBM kidney disease from the Diagnosis Procedure Combination database. *GBM* glomerular basement membrane, *KRT* kidney replacement therapy. * ≥ 1500 mg of total prednisone-converted glucocorticoid administered within 10 days of hospitalization

**Fig. 2:**
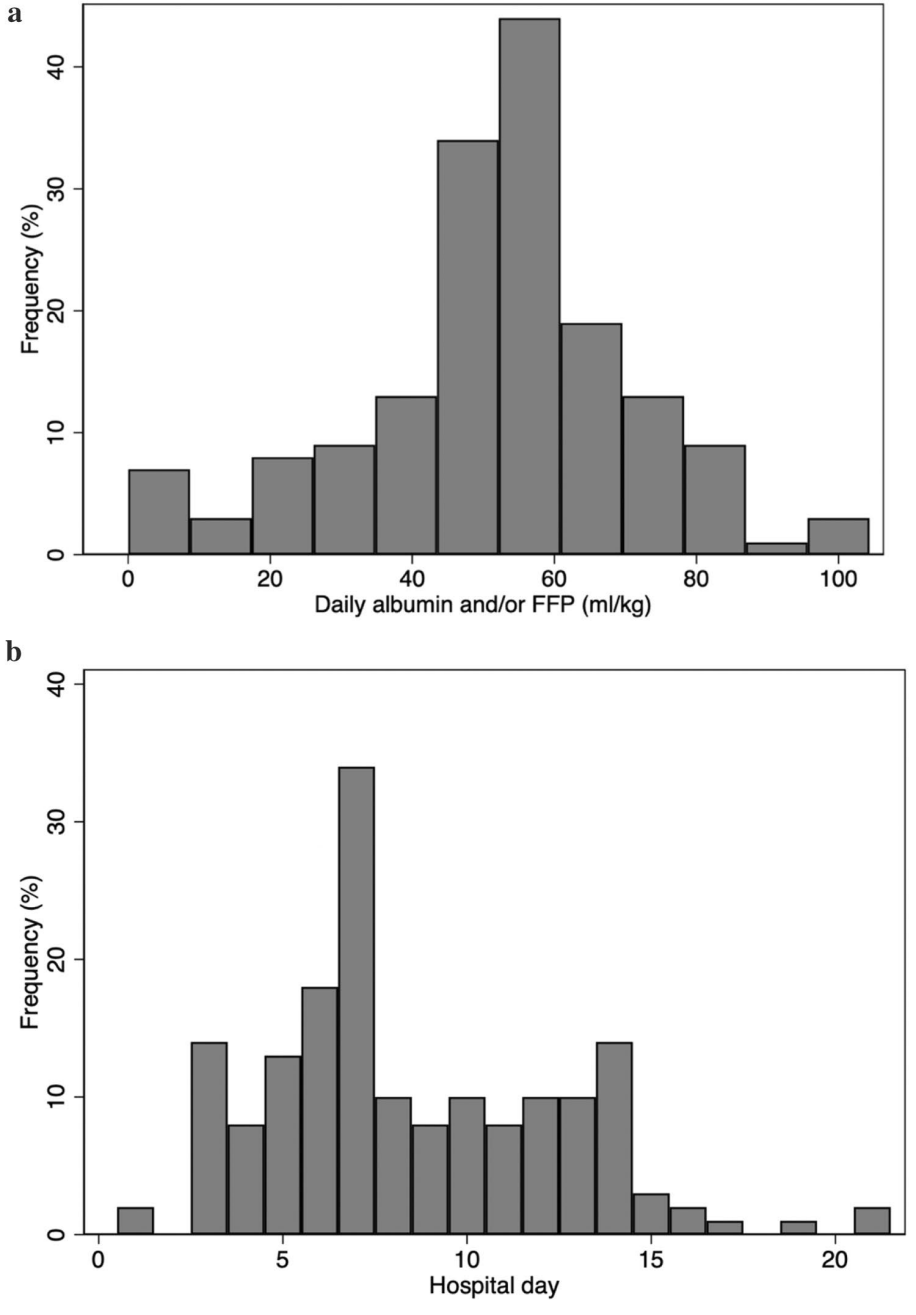
**a** Mean administered dose per day of therapeutic plasma exchange in patients with anti-GBM kidney disease (*n* = 168). **b** Total in-hospital administration days of therapeutic plasma exchange in patients with anti-GBM kidney disease (*n* = 168)

We identified 885 hospitalized patients with anti-GBM disease. A flow chart of the patient selection process is presented in Fig. 1. A total of 241 eligible patients with dialysis-dependent kidney failure without diffuse alveolar hemorrhage who received pulse glucocorticoid therapy were identified. Patient characteristics stratified by the treatment type are summarized in Online Resource 2. Thirty-four patients who received cyclophosphamide or rituximab were excluded from the primary analysis. Of the remaining 207 eligible patients from 156 hospitals, 168 received TPE within 10 days of hospitalization, and 39 did not. Ninety-two patients (44%) underwent a renal biopsy. Table 1 presents the patients’ background characteristics stratified by whether they received TPE. Overall, 69.6% of the patients were aged 60–79 years. The dose and the total number of administration days of TPE are presented in Fig. 2. The mean dose of TPE was 52.2 ml/kg/day (with a standard deviation of 19.5 ml/kg/day), and the mean number of administration days of TPE was 8.5 (with a standard deviation of 4.0). The proportion of patients who had sepsis was 19.0% (32 out of 168) among those who received TPE and 20.5% (8 out of 39 patients) among those who did not. The mean length of hospital stay was 69 days (69.3 days in those who received TPE and 68.8 days in those who did not). Patient background characteristics stratified by the use of TPE, before and after overlap weighting, are summarized in Online Resource 3. Before using overlap weights, patients who received TPE were more likely to be younger, females, and non-smokers, have a higher BMI, not have hypertension, and be hospitalized at an academic hospital than those who did not. After using overlap weights, the distribution of patient characteristics was well-balanced. The c-statistic was 0.57, and the distribution of propensity scores in the unweighted and weighted models is shown in Online Resource 4.

**Table 1:** Characteristics of eligible patients with anti-GBM kidney disease (*n* = 207)

Characteristic	With TPE* (*n* = 168)	Without TPE* (*n* = 39)	*P*-value
Age, years			
20–39	8 (4.8)	1 (2.6)	0.38
40–59	25 (14.9)	3 (7.7)	
60–79	116 (69.0)	28 (71.8)	
≥ 80	19 (11.3)	7 (17.9)	
Males	67 (39.9)	21 (53.8)	0.12
Body mass index, kg/m^2^			
< 18.5	14 (8.3)	4 (10.3)	0.57
18.5–24.9	87 (51.8)	23 (59.0)	
25.0–29.9	42 (25.0)	10 (25.6)	
≥ 30.0	13 (7.7)	1 (2.6)	
Missing	12 (7.1)	1 (2.6)	
Barthel Index score			
< 81	46 (27.4)	13 (33.3)	0.80
≥ 81	102 (60.7)	22 (56.4)	
Missing	20 (11.9)	4 (10.3)	
Current/past smoker	70 (41.7)	20 (51.3)	0.29
Academic hospital	46 (27.4)	7 (17.9)	0.24
Consciousness			
Clear	158 (94.0)	36 (92.3)	0.79
Not clear	10 (6.0)	3 (7.7)	
Charlson Comorbidity Index, mean (standard deviation)	0.88 (1.11)	0.82 (1.05)	0.66
Diabetes mellitus	42 (25.0)	10 (25.6)	0.97
Hypertension	53 (31.5)	17 (43.6)	0.09
Dyslipidemia	12 (7.1)	2 (5.1)	0.58
ANCA positivity	13 (7.7)	4 (10.3)	0.80
Use of vasopressors within 10 days of hospitalization	16 (9.5)	4 (10.3)	0.96
pRBC transfusion within 10 days of hospitalization	66 (39.3)	14 (35.9)	0.54

*Data shown as *n* (%) unless otherwise specified

*ANCA* anti-neutrophil cytoplasmic antibody, *GBM* glomerular basement membrane, *pRBC* packed red blood cell, *TPE* therapeutic plasma exchange

### Outcomes

#### Primary analysis

The overall in-hospital mortality rate was 14.0% in the 207 eligible patients. Characteristics of the patients stratified by their outcomes are presented in Online Resource 5. Those who died or continued KRT were older and had a higher Charlson comorbidity index on admission. The number of patients with each outcome event in those who received TPE vs. those who did not were as follows: in-hospital mortality, 18 (10.7%) vs. 11 (28.2%), *P* = 0.022; 28-day in-hospital mortality, 10 (6.0%) vs. 3 (7.7%), *P* = 0.708; 60-day in-hospital mortality, 13 (7.7%) vs. 5 (12.8%), *P* = 0.377; diffuse alveolar hemorrhage within 28 days of hospitalization, 21 (12.5%) vs. 7 (18.0%), *P* = 0.414; and successful discontinuation of KRT, 24 (14.3%) vs. 2 (5.1%), *P* = 0.043. Table 2 shows the results of the generalized linear regression analysis with and without overlap weighting for the outcomes in the primary analysis. Patients who received TPE had lower in-hospital mortality than those who did not; the unweighted analysis yielded a risk difference of 17.5% (95% confidence interval [CI] 2.6–32.4%; *P* = 0.022), while the weighted analysis yielded a risk difference of 17.0% (95% CI 1.5–32.5%; *P* = 0.031). We observed comparable proportions of 28-day in-hospital mortality, 60-day in-hospital mortality, diffuse alveolar hemorrhage within 28 days of hospitalization, and successful in-hospital discontinuation of KRT between the two groups in the weighted analysis (Table 2). The differences were not statistically significant.

**Table 2:** Results of generalized linear regression analysis comparing with and without therapeutic plasma exchange in those with anti-GBM kidney disease

Outcome	Model	Rate in the TPE (+) group, % (*n* = 168)	Rate in the TPE (−) group, % (*n* = 39)	Risk difference, %	95% CI		*P*-value
In-hospital mortality	Unweighted	10.7	28.2	17.5	2.6	32.4	0.022
In-hospital mortality	Weighted	11.5	28.4	17.0	1.5	32.5	0.031
28-day in-hospital mortality	Weighted	6.0	7.8	1.8	− 7.5	11.2	0.699
60-day in-hospital mortality	Weighted	8.1	13.3	5.3	− 6.6	17.2	0.385
Diffuse alveolar hemorrhage rate within 28 days of hospitalization	Weighted	12.2	18.2	6.0	− 7.3	19.4	0.377
Successful discontinuation of KRT	Weighted	11.8	5.3	6.5	− 2.4	15.3	0.151

*CI* confidence interval, *GBM* glomerular basement membrane, *KRT* kidney replacement therapy, *TPE* therapeutic plasma exchange

### Sensitivity analysis

Online Resource 6 shows the results of the sensitivity analyses. Sensitivity analyses with exposure periods of 5 or 15 days of hospitalization yielded similar results to those of the primary analysis. Other sensitivity analyses which excluded patients with missing data (*n* = 37), limited patients to those who received 3000 mg of glucocorticoids within 10 days of hospitalization (*n* = 98), and included patients who received cyclophosphamide or rituximab in addition to pulse glucocorticoid therapy (*n* = 241) yielded results similar to those of the primary analysis. The results of the survival time analyses are presented in Online Resource 7. The analyses showed the effects of TPE on survival and discontinuation of KRT that were similar to those from the main analysis.

## Discussion

In the present observational study using real-world data from a national in-patient database, the use of TPE within 10 days of hospitalization in anti-GBM disease with dialysis-dependent kidney failure without diffuse alveolar hemorrhage was associated with decreased mortality. Due to the rarity of anti-GBM disease, only one randomized trial has been conducted in the last three decades [9]. Our results show the effectiveness of TPE in improving the prognosis of the specific disease population for which there is a lack of evidence by randomized controlled trials.

We obtained a large sample of patients with anti-GBM disease, using a nationwide in-patient database. Anti-GBM disease with diffuse alveolar hemorrhage occurs more frequently in the younger population. In contrast, anti-GBM disease without diffuse alveolar hemorrhage occurs more commonly in older adults [22]. Our study focused on patients who only had involvement of the kidneys, and we found that the highest number of cases occurred among individuals in their 60s to 70s. This distribution was consistent with those in the previous studies [8, 23]. In this study, the rate of in-hospital mortality of patients who received TPE and pulse glucocorticoid therapy was 10.7%, and the mean length of hospital stay was 69.3 days before weighting. A previous single-center, single-armed, retrospective study showed a one-year mortality rate of 33.3% in patients with dialysis-dependent kidney failure who received TPE, cyclophosphamide, and glucocorticoid therapy [8]. Differences in observational periods and treatments may have caused this difference in mortality rate. However, in the previous case-series study, TPE was administered at 50 ml/kg, to a maximum of 4 L, for at least 14 days daily or until the anti-GBM antibody was undetectable [8]—similar to the dose and duration of TPE that our study population received.

Our primary analysis included the patients who received pulse glucocorticoid therapy alone as an immunosuppressive treatment. This was because the addition of cyclophosphamide and/or rituximab is not a routine treatment option in Japan, especially for dialysis-dependent patients with anti-GBM disease without diffuse alveolar hemorrhage. Based on Evidence-Based Clinical Practice Guidelines for Rapidly Progressive Glomerulonephritis 2014 by the Japanese Society of Nephrology [24], the recommendations for steroid doses and the timing of TPE use are the same as those of the Kidney Disease Improving Global Outcomes 2021 guidelines [10]. However, the timing of the administration of additional immunosuppression differs. In anti-GBM disease without diffuse alveolar hemorrhage and rapidly progressive glomerulonephritis requiring KRT, it is recommended to use a reduced dose of cyclophosphamide or, in some cases, judiciously determine if additional cyclophosphamide is necessary. This is because the chance of renal recovery is low, and the risk of other adverse events, such as leukopenia, Pneumocystis jirovecii pneumonia, bladder toxicity, and abnormal liver function, becomes higher in this setting. As shown in Online Resource 1, Japanese physicians tended to avoid using cyclophosphamide and/or rituximab in this setting. Additionally, those who received cyclophosphamide or rituximab had a high mortality rate. Although this patient group had more severe symptoms than those who received pulse glucocorticoids alone and causal inference cannot be made, this result is consistent with that in a previous report by Hirayama et al. [25]. Our sensitivity analysis, which included patients who received additional cyclophosphamide or rituximab, yielded a result similar to that of the primary analysis.

Few previous studies have focused on the effectiveness of TPE in addition to immunosuppressive therapy for anti-GBM disease—especially for anti-GBM disease with dialysis-dependent kidney failure without diffuse alveolar hemorrhage. Existing literature on patients of this disease population is confined to single-armed case reports and case series [8, 11], and current guidelines mention that the optimum role of TPE is not yet established. This is the first comparative study of 207 anti-GBM disease patients with dialysis-dependent kidney failure without diffuse alveolar hemorrhage that highlights the benefit of TPE on survival rate. While this was a retrospective observational study, the large number of patients enabled adjustment for multiple important patient backgrounds, which may have influenced effect estimates.

Following a previous review article [26], we hypothesized that the proportions of diffuse alveolar hemorrhage within 28 days of hospitalization and successful in-hospital discontinuation of KRT might differ between patients with and without TPE. Although the addition of TPE was associated with a reduction in in-hospital mortality and tended to improve these secondary outcomes, no significant differences were observed, possibly due to the small number of outcomes. A rapid removal of circulating anti-GBM antibodies by TPE may have ameliorated other complications such as congestive heart failure and immobility [3]. As we could not obtain detailed information on complications and causes of death, further research is necessary to delineate the mechanism by which TPE contributed to a better patient outcome in our study.

Our study has several limitations. First, although the overlap weighting method based on propensity scores was used to adjust for differences in baseline characteristics which may influence the decision to perform TPE, biases caused by unmeasured confounders might be present. Detailed information on the anti-GBM antibody titer level and subclass, ANCA titer level and subclass, plasma creatinine concentration, renal biopsy findings, differences in supportive care, and how rapidly the disease developed are examples of such unmeasured confounders. Second, patients who received cyclophosphamide or rituximab were excluded from the study owing to the small number of patients available in this regard. Third, we used procedures and the International Classification of Diseases 10th revision codes to define and exclude patients with diffuse alveolar hemorrhage on admission. Mild diffuse alveolar hemorrhage cases may have thus been included in the analysis. Despite these limitations, our 10-year data from the database, covering 90% of Japanese tertiary care hospitals, provides alternative evidence of our understanding of anti-GBM disease—especially when conducting large-scale randomized controlled trials is a challenge.

In conclusion, this retrospective cohort study using a national database suggests that TPE within 10 days of hospitalization may improve the in-hospital prognosis of anti-GBM disease with dialysis-dependent kidney failure without diffuse alveolar hemorrhage.

### Supplementary Information

Below is the link to the electronic supplementary material.

Supplementary file1 (DOCX 167 kb)

## Data Availability

The dataset analyzed in the current study is not publicly available because of contracts with the hospitals providing data to the database.
