# Supplementary material for: Therapeutic plasma exchange for anti-glomerular basement membrane disease with dialysis-dependent kidney failure without diffuse alveolar hemorrhage
Source: J Nephrol. 2023 Jun 24;36(8):2317–25. doi: 10.1007/s40620-023-01695-9 (PMC10638153; doi:10.1007/s40620-023-01695-9)
Supplement: Supplementary file 1 — Supplementary file1 (DOCX 167 kb) [file 40620_2023_1695_MOESM1_ESM.docx]

**Supplementary Information**

**Therapeutic plasma exchange for anti-glomerular basement membrane disease with dialysis-dependent kidney failure without diffuse alveolar hemorrhage**

*Journal of Nephrology*

Hideaki Watanabe^1*^, Hayato Yamana^2^, Akira Okada^3^, Hiroki Matsui^1^, Kiyohide Fushimi^4^, Hideo Yasunaga^1^

^1^ Department of Clinical Epidemiology and Health Economics, the University of Tokyo, Tokyo, Japan

^2^ Data Science Center, Jichi Medical University, Shimotsuke, Japan

^3^ Department of Prevention of Diabetes and Lifestyle-Related Diseases, Graduate School of Medicine, the University of Tokyo, Tokyo, Japan

^4^ Department of Health Policy and Informatics, Tokyo Medical and Dental University, Tokyo, Japan

**Corresponding author:**

Hideaki Watanabe

Email: [hwatana@m.u-tokyo.ac.jp](mailto:hwatana@m.u-tokyo.ac.jp)

| **Online Resource 1.** ICD10 codes to define sepsis. | | | | | | | | | | |
| --- | --- | --- | --- | --- | --- | --- | --- | --- | --- | --- |
| ICD-10 codes | | | | | | | | | | |
| A00 | A01 | A02 | A03 | A04 | A05 | A06 | A085 | A09 | A20 | A21 |
| A22 | A23 | A24 | A25 | A26 | A27 | A28 | A30 | A32 | A36 | A37 |
| A38 | A39 | A40 | A41 | A42 | A43 | A44 | A46 | A48 | A49 | A50 |
| A51 | A52 | A53 | A54 | A55 | A56 | A57 | A58 | A65 | A66 | A67 |
| A68 | A69 | A70 | A71 | A74 | A75 | A77 | A78 | A79 | B35 | B36 |
| B37 | B38 | B39 | B40 | B41 | B42 | B43 | B44 | B45 | B46 | B47 |
| B48 | B49 | B95 | B96 | G00 | G01 | G21 | G038 | G039 | G04 | G050 |
| G06 | G07 | G08 | I30 | I320 | I33 | I39 | I40 | I410 | I80 | J13 |
| J14 | J15 | J16 | J170 | J181 | J200 | J201 | J202 | J390 | J391 | J392 |
| J440 | J85 | J86 | K112 | K113 | K35 | K36 | K37 | K57 | K61 | K630 |
| K65 | K67 | K750 | K751 | K800 | K800 | K801 | K803 | K804 | K810 | K830 |
| L00 | L01 | L02 | L03 | L04 | L05 | L08 | M00 | M010 | M012 | M013 |
| M600 | M630 | M650 | M651 | M710 | M711 | M860 | M861 | M869 | N10 | N136 |
| N160 | N34 | N390 | N410 | N412 | N413 | N418 | N45 | N510 | N700 | N709 |
| N710 | N719 | N72 | N730 | N732 | N733 | N735 | N738 | N739 | N74 | N751 |
| N760 | N762 | N764 | O080 | O23 | O753 | O85 | O86 | O91 | R02 | R578 |
| T814 | T826 | T827 | T835 | T836 | T845 | T846 | T847 | T857 | T874 | T880 |
| ICD10: International Classification of Disease, Tenth Revision | | | | | | | | | | |

| **Online Resource 2.** Characteristics of patients with anti-GBM kidney disease, stratified by treatment type (n=241) | | | | | | |
| --- | --- | --- | --- | --- | --- | --- |
| Characteristics | | TPE (+) Cyclophosphamide (-)  Rituximab (-)  (n=168) | TPE (+) Cyclophosphamide (+)  Rituximab (-)   (n=27) | TPE (+) Cyclophosphamide (-)  Rituximab (+) (n=6) | TPE (-) Cyclophosphamide (-)  Rituximab (-) (n=39) | TPE (-) Cyclophosphamide (+)  Rituximab (-) (n=1) |
| Age, years | | | | | | |
|  | 20-39 | 8 (4.8) | 2 (7.4) | 0 (0.0) | 1 (2.6) | 0 (0) |
|  | 40-59 | 25 (14.9) | 7 (25.9) | 0 (0.0) | 3 (7.7) | 0 (0) |
|  | 60-79 | 116 (69.0) | 15 (55.6) | 5 (83.3) | 28 (71.8) | 1 (100.0) |
|  | ≥80 | 19 (11.3) | 3 (11.1) | 1 (16.7) | 7 (17.9) | 0 (0) |
| Males |  | 67 (39.9) | 14 (51.9) | 3 (50.0) | 21 (53.8) | 1 (100.0) |
| Body mass index, kg/m^2^ | | | | | | |
|  | <18.5 | 14 (8.3) | 1 (3.7) | 1 (16.7) | 4 (10.3) | 0 (0) |
|  | 18.5-24.9 | 87 (51.8) | 17 (63.0) | 3 (50.0) | 23 (58.9) | 1 (100.0) |
|  | 25.0-29.9 | 42 (25.0) | 8 (29.6) | 2 (33.3) | 10 (25.6) | 0 (0) |
|  | ≥30.0 | 13 (7.7) | 0 (0.0) | 0 (0.0) | 1 (2.6) | 0 (0) |
|  | Missing | 12 (7.1) | 1 (3.7) | 0 (0.0) | 1 (2.6) | 0 (0) |
| Barthel Index score | | | | | | |
|  | <81 | 46 (27.4) | 3 (11.1) | 0 (0.0) | 13 (33.3) | 0 (0) |
|  | ≥81 | 102 (60.7) | 19 (70.4) | 6 (100.0) | 22 (56.4) | 1 (100.0) |
|  | Missing | 20 (11.9) | 5 (18.5) | 0 (0.0) | 4 (10.3) | 0 (0) |
| Current/past smoker | | 70 (41.7) | 12 (44.4) | 2 (33.3) | 20 (51.3) | 0 (0) |
| Academic hospital | | 46 (27.4) | 6 (22.2) | 3 (50.0) | 7 (17.9) | 0 (0) |
| Consciousness | | | | | | |
|  | Clear | 158 (94.0) | 24 (88.9) | 5 (83.3) | 36 (92.3) | 1 (100.0) |
|  | Not clear | 10 (6.0) | 3 (11.1) | 1 (16.7) | 3 (7.7) | 0 (0.0) |
| Charlson Comorbidity Index,  mean (standard deviation) | | 0.88 (1.11) | 0.56 (0.89) | 1.17 (1.17) | 0.82 (1.05) | 0.00 (0.00) |
| Diabetes mellitus | | 42 (25.0) | 9 (33.3) | 1 (16.7) | 10 (25.6) | 0 (0) |
| Hypertension | | 53 (31.5) | 13 (48.1) | 2 (33.3) | 17 (43.6) | 0 (0) |
| Dyslipidemia | | 12 (7.1) | 4 (14.8) | 1 (16.7) | 2 (5.2) | 0 (0) |
| ANCA positivity | | 13 (7.7) | 1 (3.7) | 2 (33.3) | 4 (10.0) | 0 (0) |
| Use of vasopressors within 10 days of hospitalization | | 16 (9.5) | 5 (18.5) | 1 (16.7) | 4 (10.3) | 0 (0) |
| pRBC transfusion within 10 days of hospitalization | | 66 (39.3) | 13 (48.1) | 1 (16.7) | 14 (35.9) | 0 (0) |
| Mortality | | 18 (10.7) | 7 (25.9) | 2 (33.3) | 11 (28.2) | 0 (0) |
| Data shown as n (%) unless otherwise specified.  ANCA, anti-neutrophilic cytoplasmic antibody; pRBC, packed red blood cell; TPE, therapeutic plasma exchange. | | | | | | |

| **Online Resource 3.** Characteristics of eligible patients with anti-GBM kidney disease before and after using overlap weights. | | | | | | | |
| --- | --- | --- | --- | --- | --- | --- | --- |
| Characteristics | | Before using overlap weights | | | After using overlap weights | | |
|  |  | TPE (+)* | TPE (-)* | SD | TPE (+)* | TPE (-)* | SD |
| Age, years | | | | | | | |
|  | 20-39 | 4.8 | 2.6 | 11.7 | 2.7 | 2.7 | 0 |
|  | 40-59 | 14.9 | 7.7 | 22.9 | 8.6 | 8.6 | 0 |
|  | 60-79 | 69.0 | 71.8 | -6.0 | 73.0 | 73.0 | 0 |
|  | ≥80 | 11.3 | 17.9 | -18.9 | 15.8 | 15.8 | 0 |
| Males | | 40.0 | 54.0 | -28.0 | 52.0 | 52.0 | 0 |
| Body mass index, kg/m^2^ | | | | | | | |
|  | <18.50 | 8.3 | 10.3 | -6.6 | 10.1 | 10.1 | 0 |
|  | 18.5-24.9 | 51.8 | 59.0 | -14.5 | 56.8 | 56.8 | 0 |
|  | 25.0-29.9 | 25.0 | 25.6 | -1.5 | 27.3 | 27.3 | 0 |
|  | ≥30.0 | 7.7 | 2.6 | 23.6 | 2.7 | 2.7 | 0 |
|  | Missing | 7.1 | 2.6 | 21.4 | 3.1 | 3.1 | 0 |
| Barthel Index score | | | | | | | |
|  | <81 | 27.4 | 33.3 | -13.0 | 31.8 | 31.8 | 0 |
|  | ≥81 | 60.7 | 56.4 | 8.7 | 57.7 | 57.7 | 0 |
|  | Missing | 11.9 | 10.3 | 5.3 | 10.5 | 10.5 | 0 |
| Current/past smoker | | 42.0 | 51.0 | -18.4 | 50.0 | 50.0 | 0 |
| Academic hospital | | 26.0 | 17.0 | 21.6 | 19.0 | 19.0 | 0 |
| Consciousness | | | | | | | |
|  | Clear | 94.0 | 92.3 | 6.9 | 93.3 | 93.3 | 0 |
|  | Not clear | 6.0 | 7.7 | -6.9 | 6.7 | 6.7 | 0 |
| Charlson Comorbidity Index,  mean | | 88.0 | 82.0 | 5.6 | 84.0 | 84.0 | 0 |
| Diabetes mellitus | | 25.0 | 26.0 | -1.5 | 26.0 | 26.0 | 0 |
| Hypertension | | 32.0 | 44.0 | -24.9 | 42.0 | 42.0 | 0 |
| Dyslipidemia | | 7.0 | 5.0 | 8.3 | 5.0 | 5.0 | 0 |
| ANCA positivity | | 8.0 | 10.0 | 2.4 | 9.0 | 9.0 | 0 |
| Use of vasopressors within 10  days of hospitalization | | 10.0 | 10.0 | -2.4 | 10.0 | 10.0 | 0 |
| pRBC transfusion within 10 days of hospitalization | | 39.0 | 36.0 | 6.9 | 36.0 | 36.0 | 0 |
| *Data shown as % unless otherwise specified. | | | | | | | |
| Abbreviations: ANCA, anti-neutrophilic cytoplasmic antibody; pRBCs, packed red blood cells; SD, standardized difference; TPE, therapeutic plasma exchange. | | | | | | | |

**Online Resource 4:** Kernel density plots showing the distribution of propensity scores in patients who received therapeutic plasma exchange and those who did not.

(a) Before adjustment by overlap weights.


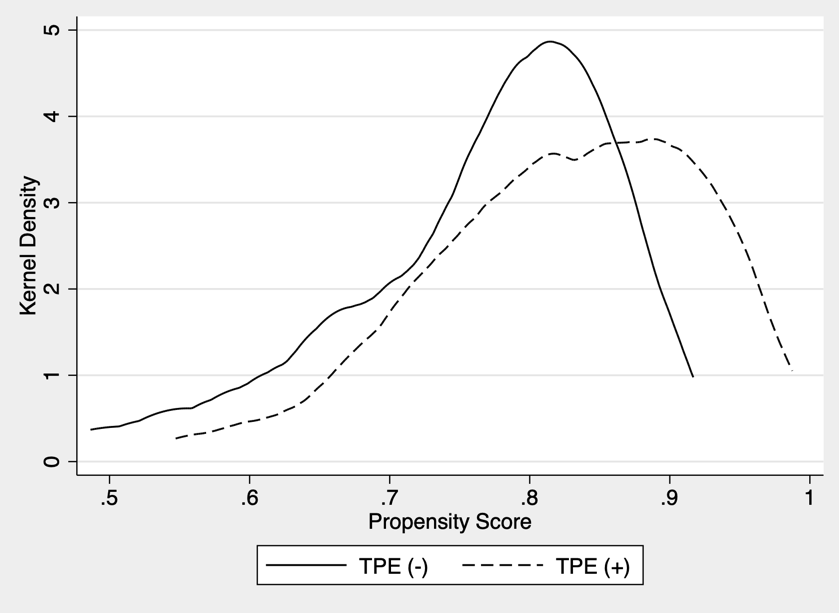


(b) After adjustment by overlap weights.


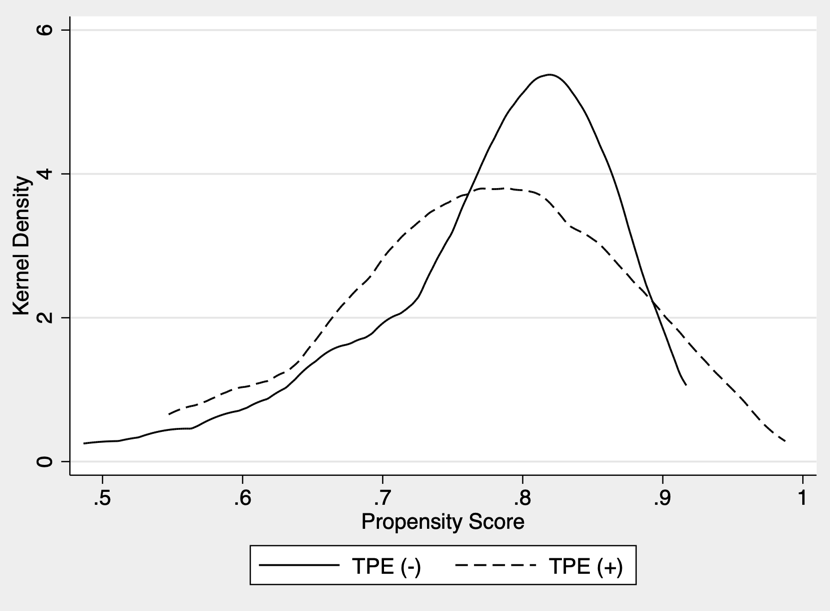


Abbreviation: TPE, therapeutic plasma exchange.

| **Online Resource 5.** Characteristics of patients with anti-GBM kidney disease, stratified by outcomes (n=207). | | | | |
| --- | --- | --- | --- | --- |
| Characteristics | | Alive & successfully  discontinued KRT  (n=26) | Alive & continued KRT (n=152) | Died  (n=29) |
| Age, years | | | | |
|  | 20 - 39 | 5 (19.2) | 4 (2.6) | 0 (0.0) |
|  | 40 - 59 | 6 (23.1) | 21 (13.8) | 1 (3.4) |
|  | 60 - 79 | 14 (53.8) | 109 (71.7) | 21 (72.4) |
|  | ≥ 80 | 1 (3.8) | 18 (11.8) | 7 (24.1) |
| Males |  | 11 (42.3) | 62 (40.8) | 15 (51.7) |
| Body mass index, kg/m2 | | | | |
|  | <18.50 | 4 (15.4) | 12 (7.9) | 2 (6.9) |
|  | 18.50 - 24.9 | 17 (65.4) | 78 (51.3) | 15 (51.7) |
|  | 25.0 - 29.9 | 4 (15.4) | 42 (27.6) | 6 (20.7) |
|  | 30.0 - | 1 (3.8) | 10 (6.6) | 3 (10.3) |
|  | Missing | 0 (0.0) | 10 (6.6) | 3 (10.3) |
| Barthel Index score | | | | |
|  | < 81 | 2 (7.7) | 44 (28.9) | 13 (44.8) |
|  | ≥ 81 | 23 (88.5) | 89 (58.6) | 12 (41.4) |
|  | Missing | 1 (3.8) | 19 (12.5) | 4 (13.8) |
| Current/past smoker | | 8 (30.8) | 68 (44.7) | 14 (48.3) |
| Academic hospital | | 10 (38.5) | 39 (25.7) | 4 (13.8) |
| Consciousness | | | | |
|  | Clear | 26 (100.0) | 143 (94.1) | 25 (86.2) |
|  | Not clear | 0 (0.0) | 9 (5.9) | 4 (13.8) |
| Charlson Comorbidity Index,  mean (standard deviation) | | 0.58 (0.76) | 0.86 (1.01) | 1.21 (1.63) |
| Diabetes mellitus | | 4 (15.4) | 38 (25.0) | 10 (34.5) |
| Hypertension | | 11 (42.3) | 54 (35.5) | 5 (17.3) |
| Dyslipidemia | | 3 (11.5) | 11 (7.2) | 0 (0.0) |
| ANCA positivity | | 1 (3.8) | 14 (9.2) | 2 (6.9) |
| Use of vasopressors within 10 days of hospitalization | | 2 (7.7) | 15 (9.9) | 3 (10.3) |
| pRBC transfusion within 10  days of hospitalization | | 7 (26.9) | 58 (38.2) | 15 (51.7) |
| TPE (+) | | 24 (92.3) | 126 (82.9) | 18 (62.1) |
| Data is shown as n (%) unless otherwise specified.  KRT, kidney replacement therapy; ANCA, anti-neutrophilic cytoplasmic antibody; pRBC, packed red blood cell; TPE, therapeutic plasma exchange | | | | |

| **Online Resource 6.** Results from the primary and sensitivity analyses. | | | | | | |
| --- | --- | --- | --- | --- | --- | --- |
| Analysis | In-hospital mortality in the TPE(+) group, % | In-hospital mortality in the TPE(-) group, % | Risk  difference, % | 95% CI | | P-value |
| Primary analysis -  Within 10 days of hospitalization | 11.5 | 28.4 | 17.0 | 1.5 | 32.5 | 0.031 |
| Sensitivity analysis -  Within 5 days of hospitalization | 8.25 | 27.3 | 19.1 | -0.3 | 38.4 | 0.054 |
| Sensitivity analysis -  Within 15 days of hospitalization | 9.1 | 20.3 | 11.2 | -3.4 | 25.8 | 0.133 |
| Sensitivity analysis -  Patients with missing data excluded | 10.5 | 26.2 | 15.8 | 0.4 | 31.9 | 0.056 |
| Sensitivity analysis -  3000 mg of glucocorticoid administered within 10 days of hospitalization | 11.0 | 27.2 | 16.2 | -7.7 | 40.0 | 0.185 |
| Sensitivity analysis -  Cyclophosphamide or rituximab included in addition to glucocorticoid pulse | 11.5 | 28.5 | 17.0 | 1.5 | 32.5 | 0.031 |
| The weighted results for in-hospital mortality are presented.  Abbreviations: CI, confidence interval; TPE, therapeutic plasma exchange. | | | | | | |

| **Online Resource 7.** Results from the weighted survival time analyses: Cox regression analysis for in-hospital mortality and competing risks analysis for death and successful discontinuation of KRT. | | | | | |
| --- | --- | --- | --- | --- | --- |
| Analysis and estimates | TPE (+) | TPE (-) | 95% CI | | P-value |
| Cox regression for in-hospital mortality | | | | | |
| Hazard ratio | 0.43 | Reference | 0.19 | 0.97 | 0.041 |
| Competing risks analysis for successful discontinuation of KRT, with death as the competing risk | | | | | |
| Subdistribution hazard ratio | 2.18 | Reference | 0.51 | 9.35 | 0.296 |
| Competing risks analysis for death, with successful discontinuation of KRT as the competing risk | | | | | |
| Subdistribution hazard ratio | 0.43 | Reference | 0.19 | 0.97 | 0.042 |
| Abbreviations: CI, confidence interval; KRT, kidney replacement therapy; TPE, therapeutic plasma exchange. | | | | | |
